# Conductive Chitosan–Graphene Oxide Scaffold with Applications in Peripheral Nerve Tissue Engineering

**DOI:** 10.3390/polym17172398

**Published:** 2025-09-02

**Authors:** Andreea-Isabela Lazăr, Aida Șelaru, Alexa-Maria Croitoru, Ludmila Motelica, Ovidiu-Cristian Oprea, Roxana-Doina Trușcă, Denisa Ficai, Dănuț-Ionel Văireanu, Anton Ficai, Sorina Dinescu

**Affiliations:** 1 Department of Science and Engineering of Oxide Materials and Nanomaterials, Faculty of Chemical Engineering and Biotechnologies, National University of Science and Technology Politehnica Bucharest, 1-7 Gh. Polizu St., 011061 Bucharest, Romania; lazarisabela1997@gmail.com (A.-I.L.); alexa_maria.croitoru@upb.ro (A.-M.C.); truscaroxana@yahoo.com (R.-D.T.); 2 National Centre for Micro- and Nanomaterials, National University of Science and Technology Politehnica Bucharest, 313 Independence Boulevard, 060042 Bucharest, Romania; motelica_ludmila@yahoo.com (L.M.); ovidiu.oprea@upb.ro (O.-C.O.); denisaficai@yahoo.ro (D.F.); 3 National Centre for Food Safety, National University of Science and Technology Politehnica Bucharest, 313 Independence Boulevard, 060042 Bucharest, Romania; 4 Department of Biochemistry and Molecular Biology, Faculty of Biology, University of Bucharest, 91-95 Independence Boulevard, 050095 Bucharest, Romania; aida.selaru@bio.unibuc.ro (A.Ș.); sorina.dinescu@bio.unibuc.ro (S.D.); 5 Department of Inorganic Chemistry, Physical Chemistry and Electrochemistry, Faculty of Chemical Engineering and Biotechnologies, National University of Science and Technology Politehnica of Bucharest, 1-7 Gh. Polizu St., 011061 Bucharest, Romania; di_vaireanu@yahoo.co.uk; 6 Academy of Romanian Scientists, 3 Ilfov St., 050045 Bucharest, Romania; 7 Research Center for Advanced Materials, Products and Processes, National University of Science and Technology Politehnica Bucharest, 060042 Bucharest, Romania; 8 Technical Sciences Academy of Romania, 26 Dacia Boulevard, 030167 Bucharest, Romania; 9 Research Institute of the University of Bucharest (ICUB), 90 Panduri Road, 050663 Bucharest, Romania

**Keywords:** tissue engineering, chitosan, crosslinking, electric conductivity, glutaraldehyde, composite materials, graphene-oxide, neuronal applications

## Abstract

This study aimed to develop a novel biomaterial for neural tissue regeneration by combining chitosan (CS), a natural polymer, with graphene oxide (GO) at concentrations of 3%, 6%, and 9%. The homogeneity, conductivity, three-dimensional characteristics, and ability to support cell viability of the composite materials were systematically evaluated. Fourier-Transform Infrared (FTIR) spectroscopy confirmed the successful incorporation of GO into the CS matrix, while UV-Vis and photoluminescence (PL) spectrometry revealed modifications in the optical properties with increasing GO content. Thermogravimetric analysis (TG-DSC) demonstrated improved thermal stability of the composites, and swelling tests indicated enhanced water absorption capacity. Although some agglomerates were observed, the homogeneity was reasonable at both macroscopic and microscopic level (optical visualization–FTIR and electron microscopy). The composite films exhibited promising physical and electrochemical properties, highlighting their potential for neural tissue engineering applications. Their biological activity was assessed by culturing neuronal cells on the CS-GO scaffolds. Results from MTT, LDH, and LIVE/DEAD assays demonstrated excellent cell viability, moderate-to-good cell attachment, and the promotion of intercellular network formation. Among the tested formulations, the CS-GO 6% scaffold showed the most favorable biological response, with a significant increase in SH-SY5Y cell viability after 7 days (*p* < 0.05) compared to the CS control. LIVE/DEAD imaging confirmed enhanced cell attachment and elongated morphology, while the LDH assay indicated minimal cytotoxicity. Notably, a critical threshold was identified between 6% and 9% GO, where conductivity increased by approximately 52-fold. Future studies should focus on optimizing the composite parameters, loading them with specific biologically active agents and thus targeting specific neuronal applications.

## 1. Introduction

Neuronal tissue regeneration is particularly challenging, and the development of innovative scaffolds aims to facilitate the repair of damaged or injured neural tissues. Treating nerve injuries often involves using an autologous nerve graft to bridge the gap. However, nerve autografts have several drawbacks, including the necessity of impairing a healthy, functional nerve as well as an additional intervention to harvest it [1]. With the rapid progress in regenerative medicine, engineered nerve guidance conduits (NGCs) show the potential to address these limitations and enhance neural recovery. However, developing nerve grafts that fully meet clinical demands remains a significant challenge [2].

Despite their limited electrical conductivity, various materials are being explored as scaffolds for nerve regeneration. Preclinical studies frequently employ natural biopolymers due to their biocompatibility and support for cell attachment and growth. These include structural proteins such as collagen, gelatin, silk, and glycosaminoglycans or polysaccharides like hyaluronic acid, alginate, and CS [3]. Natural polymers, in particular, are utilized due to their excellent biocompatibility, adjustable biodegradability, and specific bioactive properties. The majority of synthetic materials researched for nerve scaffolds are polyester-based; however, other polymers, such as polyethylene glycol (PEG), are being investigated for their regenerative properties. However, its regenerative effects are not intrinsic; they are typically observed when PEG is functionalized with bioactive moieties such as peptides, growth factors, or adhesion molecules that can actively guide axonal regeneration [4].

CS is often used on its own or combined with other materials and is a widely recognized polymer in tissue engineering and regenerative medicine, valued for its biocompatibility, biodegradability, non-toxic nature, and beneficial antibacterial properties [5]. It is often cited in the literature as a primary component in novel tissue-engineered nerve conduits designed to bridge peripheral nerve gaps [6,7,8]. By attaching various functional groups, the hydrophilic–hydrophobic ratio as well as the electric charge of CS support can be controlled. These modifications contribute to CS’s status as a relatively unique, highly tunable biopolymer [9]. Due to these beneficial properties, a CS tube received FDA approval in 2015 for peripheral nerve repairs up to 1 cm. Additionally, Gu et al. successfully used CS-based scaffolds to repair a 30 mm long defect in the human median nerve [10]. Ongoing intensive preclinical research is focused on developing more advanced designs and functionalities to extend the gap that can be effectively supported for regeneration [11]. Due to its tunable mechanical properties, the degradation rate of CS-based materials can be customized to match the pace of nerve healing. Furthermore, it can serve as a drug delivery system, releasing therapeutic agents at the injury site to enhance regeneration [12].

The methods for modifying the polymer include chemical crosslinking, the addition of plasticizers [13], the creation of new polymer networks, and the incorporation of organic and inorganic nanoparticles such as carbon [14], silver [15,16], or gold [15,17] nanoparticles, bioglass [18], etc. Among these approaches, the combination with GO is one of the most widely researched areas [19,20,21]. Recent research demonstrates that the synergistic interaction between CS and GO exhibits excellent biocompatibility in both in vitro and in vivo settings [22,23,24], along with angiogenic and cell growth-promoting effects [25,26], antimicrobial activity [27,28], electrical conductivity [29], and superior adsorption capabilities [30,31], among other properties. Although GO is frequently employed in neural tissue engineering, it is not inherently highly conductive. Its oxygen-containing functional groups disrupt the π-conjugated network, significantly reducing electrical conductivity [32]. In contrast, conductive polymers such as polypyrrole (PPy) or polyaniline (PANI), and their composites with GO or rGO, can enhance conductivity by several orders of magnitude. For instance, GO/PPy composites may achieve conductivities on the order of ~6 S/cm that remain stable in physiological conditions over weeks, while yielding strong mechanical performance suitable for nerve repair scaffolds [33]. Similarly, biodegradable polymer composites containing ~20% PPy have shown conductivity increases of nearly 300% compared to the base polymer alone [34].

Despite this, GO-based scaffolds offer unique advantages, including good hydrophilicity, abundant reactive sites for functionalization, and excellent biocompatibility when combined with natural polymers like CS. Moreover, moderate conductivity levels (as demonstrated in CS-GO scaffolds with conductivities of ~2.8 mS/cm) have been sufficient to support neural cell adhesion, neurite outgrowth, and functional recovery in vivo [35]. Therefore, while GO alone may not match the conductivity of conductive polymer-enhanced composites, it provides a balanced platform for scaffold design with appropriate electrical, biological, and processing features.

In addition, it is worth noting that previous studies have shown that CS materials used in nerve repair are often modified with different degrees of acetylation to regulate their biodegradation rate [36,37,38]. CS is also frequently combined with other materials, such as polyglycolic acid (PGA) [39,40,41], polylactic acid (PLA) [42,43], or other commonly used polymers [44]. Some formulations use blends of CS with multiple polymers, such as a combination of PGA and PLA. By attaching various functional groups, the hydrophobic, cationic, and anionic properties of CS can be controlled [45]. CS-based materials can also be enhanced by modifying their surfaces to interact with regenerating neural tissues. For instance, extracellular matrix glycoproteins like laminin [7] or laminin-derived peptides [6,46], as well as collagen [47], can be applied to the surface of CS-based materials to enhance their compatibility with neural tissues. Moreover, neurotrophic factors such as NGF, GDNF, CNTF, NT-3, or NT-4/5 can be integrated [48,49,50,51,52,53]. The interior surfaces of these guidance channels can be designed to support the seeding of cells that aid in regeneration, such as aligned Schwann cells [54] or various types of stem cells [55]. Lastly, electrospinning technology is employed to add structured nano- or microfibers to the surface of the CS-based materials for additional functionality [56].

This study explores the potential of CS-based scaffolds incorporated with GO for peripheral nerve regeneration, focusing on their biocompatibility, electrical conductivity, and cell viability. Most prior works have focused on either morphological or biological characterization, without systematically correlating GO concentration with both electrical and biological performance in a controlled gradient. Moreover, the identification of a critical GO concentration that optimizes both conductivity and neural cell compatibility remains underexplored. While increased GO loading can improve conductivity, it may also introduce cytotoxicity or structural instability, which limits its therapeutic application.

To address this gap, the present study investigates the effect of varying GO concentrations (3%, 6%, and 9%) within CS-based scaffolds, aiming to identify the optimal formulation that balances electrical properties, structural integrity, and neuronal biocompatibility. Through a combination of physicochemical characterization (FTIR, SEM, swelling, thermal stability, and conductivity) and in vitro biological assays (MTT, LDH, and LIVE/DEAD using SH-SY5Y cells), this work aims to provide a quantitative threshold for GO concentration that enhances scaffold performance without compromising safety (a critical step for advancing CS-GO scaffolds toward neuroregenerative applications). Furthermore, the study aims to improve nerve repair outcomes and address the limitations of traditional autografts in clinical applications.

## 2. Materials and Methods

### 2.1. Materials

GO was synthesized using Hummer’s method [57]. The CS used in this study was purchased from Sigma-Aldrich with a medium molecular weight (190,000–310,000 Da), a viscosity ranging from 200 to 800 cP, and a degree of deacetylation between 75% and 85%. Glutaraldehyde (GA; OHC(CH_2_)_3_CHO) was also obtained from Sigma-Aldrich (Merck, Burlington, MA, USA) as a 25% aqueous solution (*w*/*w*). Distilled water was used throughout the study. Glycerol (99.0–101.0% purity, alkalimetric) was supplied by Honeywell, while glacial acetic acid (CH_3_COOH) was sourced from Chimreactiv S.R.L., with a stated purity of 99.84%. Sodium hydrogen phosphate (Na_2_HPO_4_, min. 99% purity) and sodium phosphate (Na_3_PO_4_, min. 99% purity) were acquired from Reactivul București. Tween 80 (polyoxyethylene sorbitan monooleate) was purchased from Sigma-Aldrich. The human neuroblastoma cell line SH-SY5Y (CRL-2266, ATCC) was used as an in vitro model for evaluating the biocompatibility of CS-GO scaffolds. All reagents utilized in this study were used without further purification.

### 2.2. Synthesis of CS-GO Scaffolds

**Figure 1:**
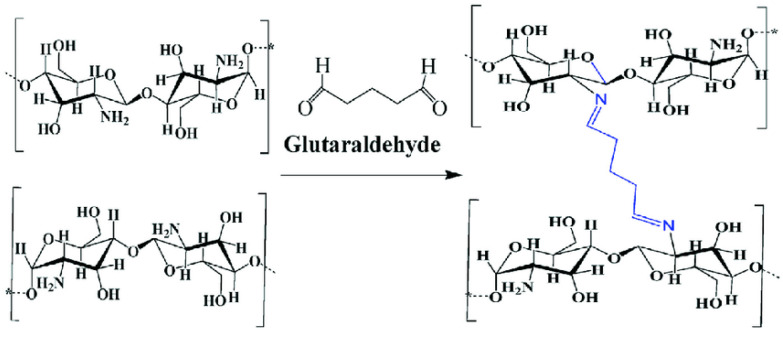
Adding GA facilitates the formation of bonds between CS molecules, resulting in a cross-linked network structure [61]. Adapted from Elsevier, 2020. The asterisk (*) denotes the continuation of the CS polymer chain.

CS was activated prior to crosslinking by treating it with an acid solution, a critical step for exposing reactive sites on the CS molecules and enhancing their crosslinking potential [58]. A 30 mg/mL CS solution was prepared in 6.0% acetic acid, followed by the addition of 1.0 mL glycerin to increase the viscosity. The mixture was stirred continuously using a magnetic stirrer at room temperature for 24 h to ensure homogeneity The CS solution was combined with varying amounts of GO (3%, 6%, and 9%). The required amount of GO was first mixed with 10 mL of CS solution by manual grinding to form a uniform paste, followed by sonication to ensure additional disaggregation of the GO clusters. Subsequently, 0.1% (*w*/*v*) Tween 80 was added to the CS-GO mixture to further enhance dispersion. To the homogenized mixture, 0.15% glutaraldehyde (GA) was added to induce crosslinking. GA is a bifunctional crosslinking agent, meaning that it has two reactive groups that can form covalent bonds with amino groups present in CS [59]. The reaction was carried out in an aqueous solution, where GA reacted with CS to form covalent bonds between the CS molecules. This led to the formation of a cross-linked network (Figure 1), which significantly enhances the mechanical strength, stability, and biocompatibility of CS, thereby making it suitable for various biomedical applications [60].

-NH_2_ (CS) + CHO-CHO (glutaraldehyde) → -NH-CH_2_-CHOH-CH_2_-NH- (cross-linked CS)

A 10 mL aliquot of each mixture was cast into transparent glass Petri dishes and immersed in 10 mL Na_2_HPO_4_/Na_3_PO_4_ buffer. The solutions were left undisturbed at room temperature for 48 h to allow solvent evaporation and the formation of smooth, thin films. Pure CS scaffolds were prepared under identical conditions to serve as controls. The resulting films were thoroughly washed three times with distilled water to remove any unreacted GA and other by-products, then carefully dried, and peeled from the Petri dishes for further characterization (Table 1).

### 2.3. Fourier-Transform Infrared Spectroscopy

The presence of functional groups and interactions between various components of the composite films were investigated using FTIR in the wavenumber range 4000–400 cm^−1^. The spectra were recorded with a Nicolet iS50 FTIR spectrometer (Nicolet City, MA, USA), equipped with a DTGS detector, at a resolution of 4 cm^−1^, by averaging 32 scans. All spectra were registered in attenuated total reflectance (ATR) mode using a diamond crystal.

To obtain information about the spatial distribution of the components, FTIR 2D maps were recorded with an FTIR microscope Nicolet iS50R (Nicolet City, MA, USA), with DTGS detector, in the wavenumber range 4000–500 cm^−1^.

### 2.4. UV-Vis Spectroscopy

A JASCO V560 spectrophotometer (JASCO Inc., Easton, PA, USA) was used to measure the UV-Vis spectra. The device was equipped with a 60 mm integrating sphere (ISV-469) and a film holder for the samples. The spectra were recorded with a speed of 200 nm min^−1^, in the range of 200–900 nm.

The opacity values were calculated as A_600_/x = −logT_600_/x, where A_600_ is the absorbance at 600 nm, T_600_ is the fractional transmittance at 600 nm, and x is the film thickness in mm. A higher opacity value indicated that the film was less transparent [62].

### 2.5. PL Spectrometry

A Perkin Elmer (Waltham, MA, USA) LS55 spectrometer was used to measure the photoluminescence spectrum (PL). A Xe lamp served as a UV light source at ambient temperature. Fluorescence was measured in the range 350–600 nm. The spectra were recorded with a scan speed of 200 nm min^−1^, excitation and emission slits of 10 nm, and a 350 nm cut-off filter. An excitation wavelength of 320 nm was used.

### 2.6. Thermal Analysis

For thermal analysis, TG-DSC, approximately 10 mg from each sample was placed in an open alumina crucible in a Netzsch (Selb, Germany) STA 449C Jupiter apparatus. It was then heated from room temperature up to 900 °C, with 10 K∙min^−1^, under a flow of 50 mL∙min^−1^ dried air. An empty alumina crucible was used as a reference.

### 2.7. Swelling Capacity, pH, and Conductivity Variation

The samples were weighed (W_0_) and then were immersed in 200 mL phosphate-buffered saline (PBS) (pH = 7.4) to facilitate swelling. For each film, three successive measurements were taken, and their average value was used as a reference. The samples were weighted at specific intervals: 5 min, 10 min, 30 min, 60 min, 4 h, 6 h, 18 h, 24 h, and 48 h. The swelling degree (D) was calculated using Equation (1):

D = (W_t_ − W_0_)/W_0_ (1)

For each sample, the swelling, pH, and conductivity were measured at the predetermined time intervals. All experiments were carried out in triplicate (*n* = 3). Data are presented as mean ± standard deviation (SD). At each time point, the experimental groups (CS–3% GO, CS–6% GO, and CS–9% GO) were compared with the CS control group using two-sample *t*-tests. Statistical significance was defined as *p* < 0.05 (*), *p* < 0.01 (**), and *p* < 0.001 (***). Data were analyzed under the assumption of approximate normal distribution, in line with common practice in biomaterials studies.

### 2.8. Electrochemical Conductivity Measurements of CS-GO

CS-GO films (3%, 6%, and 9%) were deposited onto stainless steel plates, to provide a conductive support. To ensure accurate electrical resistance measurements, the four-electrode method was employed, suitable for low-resistance materials. A RC3563 four-electrode instrument (Changzhou Chuangkai Electronic Co., Ltd., Changzhou, Jiangsu, China) adapted for this purpose was used, with two of the electrodes in contact with the stainless-steel plate and the other two attached directly to the deposited CS-GO film. The resulting assembly was placed between the jaws of a digital micrometer (Mitutoyo Corporation, Kawasaki, Japan) capable of measuring layer thickness with micrometer resolution. The micrometer’s dynamometric screw applies constant pressure on the deposited layer, regardless of the nature of the deposit, ensuring identical conditions for all evaluations. In this configuration, one electrode pair applies a current through the deposited material, while the other measures the resulting voltage. The electrical resistance (R) of the materials can be expressed in terms of conductivity (λ), layer thickness (*d*), and cross-sectional area (S) using Equation (2):

(2) $$R=\frac{1}{\lambda }\frac{d}{S}$$

The initial thickness of the steel plates, L_0_, was measured before the deposition of the materials using the same digital micrometer. This allowed us to calculate the conductivity of the evaluated sample based on the total thickness of the array, L_1_, and the thickness of the steel plates, L_0_.

The thickness of the deposited layer was determined using Equation (3):

d _deposited layer_ = L_1_ − L_0_ (3)

### 2.9. SEM Analysis

The investigation of the samples was carried out using the scanning electron microscope QUANTA INSPECT F50, equipped with a field emission electron gun—FEG (field emission gun)—with a resolution of 1.2 nm and an energy dispersive X-ray spectrometer (EDS) with a resolution of MnK of 133 eV. The surface and cross-sections of the CS samples were coated with a thin layer of gold prior to the examination.

To investigate the surface samples for good electrical conductance, the samples were metallized for 60 s with gold.

### 2.10. Biocompatibility Assessment of SH-SY5Y Cell Culture in Contact with CS-GO Scaffolds

A cell–scaffold system was obtained by seeding an initial density of 4.5 × 10^4^ cells/cm^2^ on the materials’ surface. These systems were maintained in specific culture media, a mixture of 1:1 minimum essential medium eagle (MEM) and F12 medium supplemented with 10% fetal bovine serum (FBS) and 1% antibiotic. The CS-GO/SH-SY5Y systems were then incubated for one week under standard culture conditions (37 °C, 5% CO_2_, and 95% humidity). Biocompatibility assays were performed at 3 and 7 days post-seeding. A plain CS scaffold served as a control for these assays.

The quantitative assessment of cell viability and proliferation was evaluated using the 3-(4,5-dimethylthiazol-2-yl)-2,5-diphenyltetrazolium bromide (MTT) spectrophotometric assay. Cells cultured on CS-GO scaffolds were treated with a 1 mg/mL^−1^ solution of MTT for 4 h under standard culture conditions. The resulting formazan crystals were dissolved in isopropanol, and the final solution was measured at 550 nm using a FlexStation3 spectrophotometer (Molecular Devices, San Jose, CA, USA).

The cytotoxic effect of the composites upon SH-SY5Y cultures was assessed by spectrophotometrically determining the lactate dehydrogenase (LDH) levels in the culture media using an “In vitro toxicology assay kit lactate dehydrogenase-based” TOX7 Kit (Sigma-Aldrich Co., Steinheim, Germany). The assay was carried out following the manufacturer’s instructions, and the final solution was measured at 490 nm using a FlexStation3 spectrophotometer (Molecular Devices, San Jose, CA, USA).

To qualitatively assess the cellular behavior of SH-SY5Y cultures in contact with CS-GO scaffolds, a Live/Dead Kit (Invitrogen, Life Technologies, Foster City, CA, USA, L3224) and fluorescence microscopy were used. The method enabled the simultaneous visualization of live cells, dyed green with calcein AM, and the nuclei of dead cells, stained red with ethidium bromide. The examination of the samples was performed using a laser-scanning confocal microscope (Nikon A1/A1R Confocal Laser Microscope System, Tokyo, Japan), and images were analyzed with the corresponding software.

## 3. Results and Discussion

### 3.1. FTIR Spectroscopy

**Figure 2:**
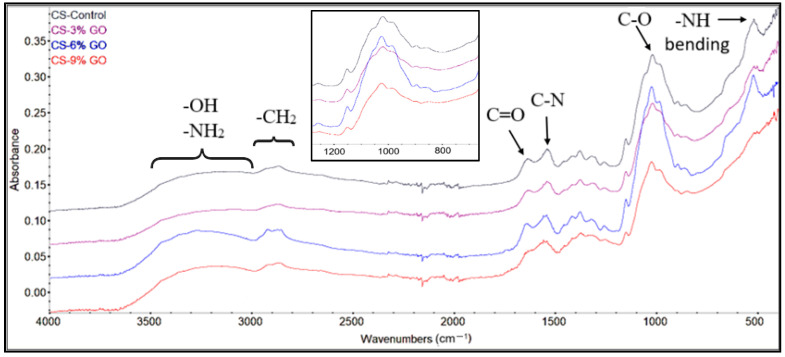
FTIR spectra for CS, CS-3% GO, CS-6% GO, and CS-9% GO.

FTIR spectroscopy analysis provided detailed insights into the functional groups present in the CS and CS-GO materials by measuring their infrared (IR) radiation absorption. Based on the IR spectrum of CS (Figure 2), the main characteristic peaks were clearly identified and found to be consistent with those reported in previous studies [63,64,65]. The broad band in the region of 3500–3000 cm^−1^ corresponded to –OH and –NH_2_ stretching, while the band between 2800 and 3000 cm^−1^ was attributed to CH_2_ stretching modes. The presence of the C=O stretching vibration of amide I was confirmed by the peak at 1635 cm^−1^, and the band at 1541 cm^−1^ was assigned to the C–N stretching vibrations of amide III. Additionally, the band observed at 1019 cm^−1^ corresponded to C–O stretching vibrations, while the peak at 518 cm^−1^ was associated with out-of-plane NH bending, as also observed in the CS FTIR spectra.

The FTIR spectra for GO, as detailed in our previous study [66], provided further context for the interaction of GO with CS. A combination of both CS and GO characteristic peaks was observed in the FTIR spectra of CS-GO materials. Moreover, a strong shift in the peak from 1541 cm^−1^ of CS to 1577 cm^−1^ (observed for CS-9% GO) was observed, due to the interaction between the amino groups from CS surface with the hydroxy groups on the GO surface. Similarly, a visible shift of 3 to 7 cm^−1^ of the bands between 1000 and 1050 cm^−1^ was observed, highlighting the interaction between GO and CS. These interactions were important, as the potential toxicity of GO could thus be reduced by coating the GO. As the GO concentration increased further, the peak from 1019 cm^−1^ (observed for CS-3% GO) shifted to 1026 cm^−1^ (observed for CS-9% GO). This modification was attributed to the saturation of oxygen-containing groups on GO, which limited their interaction with the CS surface. Furthermore, the absence of the peak around 1700 cm^−1^, typically associated with C=O stretching vibrations in the carbonyl and carboxyl groups of GO, was attributed to the GO/CS mass ratio, which rendered the peak too weak to detect. The entrapment of the GO sheets within the CS matrix, as well as the strong interactions between CS and GO [67,68], played an essential role in limiting the development of the “foreign body effect”, with GO being mostly covered by CS.

### 3.2. FTIR Microscopy

**Figure 3:**
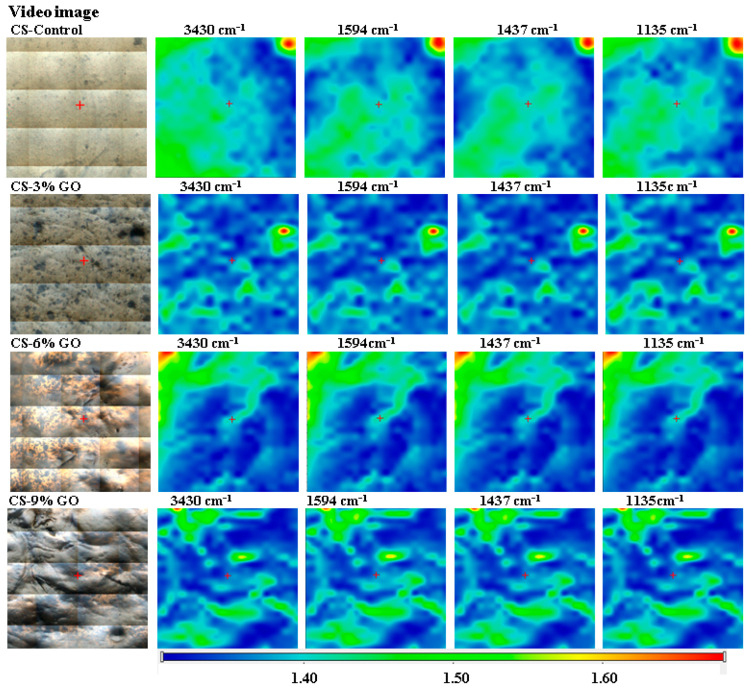
Hyperspectral FTIR imaging of CS and CS-GO films with different GO concentrations: distribution of key functional groups at 3430 cm^−1^ (O–H/N–H), 1594 cm^−1^ (C=C), 1437 cm^−1^ (C–N), and 1135 cm^−1^ (C–O). The intensity scale represents absorbance levels.

The video and FTIR maps in Figure 3 were recorded at the characteristic peaks for CS and CS-GO films with 3%, 6%, and 9% GO concentrations. The incorporation of GO resulted in changes in peak intensity and slight shifts, particularly at 3430 cm^−1^, indicating enhanced hydrogen bonding between GO’s oxygen-containing groups and CS’s hydroxyl and amine functionalities. Additionally, the increase in intensity and the shift in the main band associated with the COH bands of the CS (from ~1019 to over 1025 cm^−1^) in the presence of higher GO content reflected the interactions between the GO and CS matrix. These spectral modifications confirmed the successful integration and the interaction of GO with the CS structure.

FTIR microscopy was a suitable technique to evaluate the microstructure of the composite at a level between visual/optical and electron microscopy levels. Based on the images recorded at the four wavelengths, no considerable differences were observed, indicating that GO did not form agglomerates of tens of hundreds of µm in diameter, which would have been clearly observed in FTIR microscopy.

### 3.3. UV-Vis Spectroscopy

The yellowish-tinted CS transparent films presented an absorption maximum around 350 nm. The asymmetric absorption band presented a long tail in the visible domain, in the violet–blue region, which was responsible for the yellowish appearance. This color was specific for the CS-based films [69].

**Figure 4:**
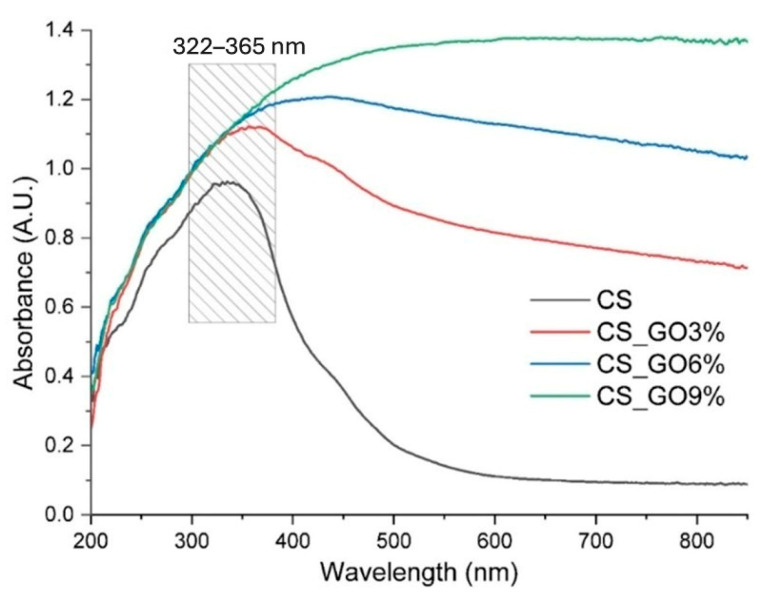
UV-Vis spectra of CS-based films.

The addition of GO to the polymer matrix changed the color to black, and as a consequence, the films strongly absorbed light on the entire visible domain. The films became darker as the GO quantity increased from 3% to 9%, which was also indicated by the increasing absorbance for the CS-GO 9% film (Figure 4).

The film thickness and opacity values are given in Table 2. It was observed that while the thickness marginally increased with GO incorporation, the opacity increased by an order of magnitude starting from the addition of 3% GO. Increasing the GO content to 6% significantly enhanced the opacity, while further increase to 9% had a smaller influence on an already opaque composite film.

### 3.4. PL Spectrometry

**Figure 5:**
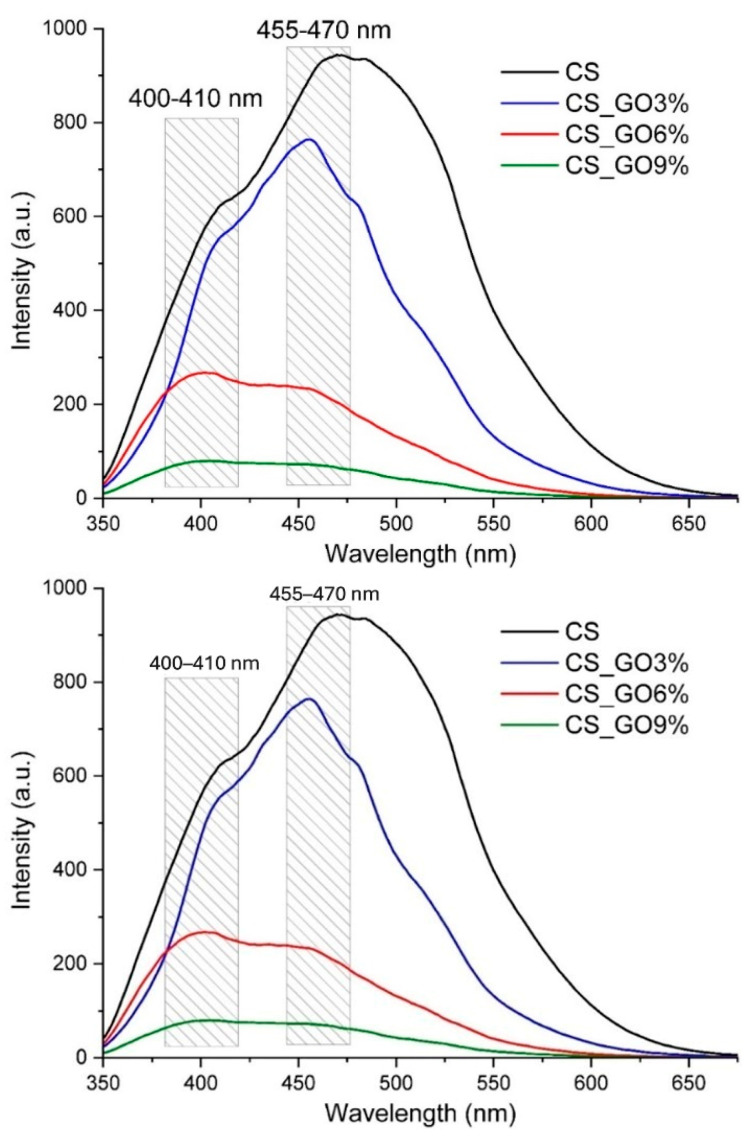
The fluorescence (PL) spectra of CS-based films.

The CS film exhibited a strong blue emission fluorescence (Figure 5) as all polysaccharides, like alginate or cellulose [62,69,70]. The emission band, with multiple peaks and a tail towards longer wavelengths, covered the violet–green region of the spectrum and indicated the possible multiple origins of the emitted photons. By adding GO to the CS-based films, the intensity of the fluorescent emission decreased drastically. The band from the blue–green region diminished more than the violet emission, indicating possible covalent interactions between GO and CS polymer chains. This quenching effect may be attributed to the introduction of non-radiative pathways by GO, likely through surface conduction mechanisms that facilitate the recombination of excited electrons and corresponding holes.

### 3.5. Thermal Analysis

**Figure 6:**
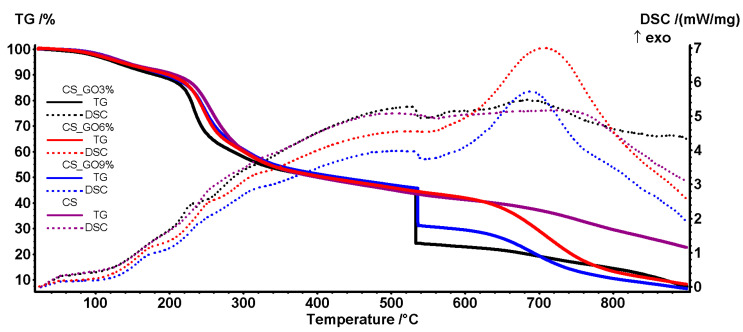
The thermal analysis (TG and DSC curves) for CS and CS-GO samples.

The thermal behavior of all films up to 200 °C was similar, with a mass loss of ~10% representing the elimination of water molecules (Figure 6). The residual moisture was eliminated during the first stage, as indicated by an endothermic effect on the DSC curve. Above 100 °C, the samples began to degrade due to the loss of water by polymer chain dehydration.

Fragmentation of the polymer backbone occurred above 200 °C, along with partial oxidation. Between 400 °C and 600 °C, GO also underwent oxidation reactions, explosively, as reported in [71]. In the CS-GO 6% sample, the oxidation reaction occurred more gradually, suggesting an optimized bonding between the polymer chains and GO, which led to a better stabilization of the GO structure. After 600 °C, the combustion of the carbonaceous residual mass took place, accompanied by a strong exothermic effect on the DSC curve. Additional details are provided in Supplementary Materials.

Table 3 presents the main numeric data from the thermal analysis, including the temperatures corresponding to mass losses of 5%, 10%, or 15% for each sample.

### 3.6. Swelling Behavior

**Figure 7:**
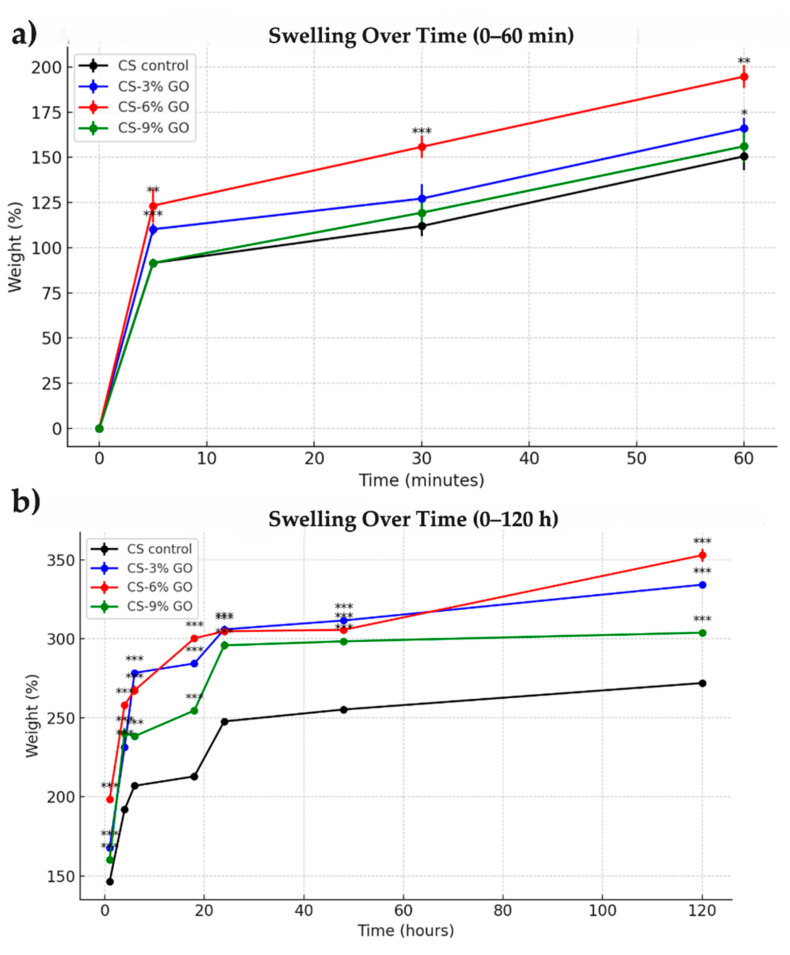
Swelling behavior of CS-based films over short- (**a**) and long- (**b**) term periods. Mean and standard deviation (SD) were calculated for each group at each time point. Statistical analysis was performed using one-way ANOVA, followed by Tukey’s post hoc test to compare groups. Statistical significance was denoted as follows: *p* < 0.05 (*); *p* < 0.01 (**); *p* < 0.001 (***). All experiments were performed in triplicate (*n* = 3).

For all swelling evaluations, we considered samples of 0.46–0.65 g. Within the first 5 min, all samples showed a noticeable increase in swelling, with weights rising up to 125% *w*/*w* (Figure 7).

As shown in Figure 7a, all samples exhibited a rapid increase in swelling within the first 5 min, followed by a slower, more gradual increase over the remaining duration. The control CS control demonstrated the lowest swelling capacity, reaching 146% at 60 min. In contrast, scaffolds containing GO exhibited enhanced swelling behavior, with the 9% GO sample showing the highest swelling ratio (160%). The addition of GO appears to improve water uptake, likely due to the hydrophilic nature and high surface area of GO, which enhanced fluid absorption.

As illustrated in Figure 7b, swelling continued to increase over time for all formulations, but the rate of increase diminished significantly after the first 24 h, indicating stabilization. The CS–9% GO scaffold consistently exhibited the highest swelling capacity, reaching 304.62% at 120 h. The CS-6% GO scaffold followed closely with 352.73%, while the CS–3% GO and control scaffolds reached lower plateau values of 332.61% and 272%, respectively.

These results confirm that higher GO content promoted sustained water uptake, likely due to improved scaffold porosity and hydrophilicity.

### 3.7. In Vitro Evaluation of the Grafting Materials

This experiment aimed to evaluate the impact of introducing CS and CS-GO scaffolds into a PBS solution, focusing on the pH and conductivity variations over time. This was intended to simulate the interaction between the materials and a physiological solution, particularly in terms of their potential to alter the PBS environment, which is critical for various biological processes, including neuroregeneration.

#### 3.7.1. pH Variation

**Figure 8:**
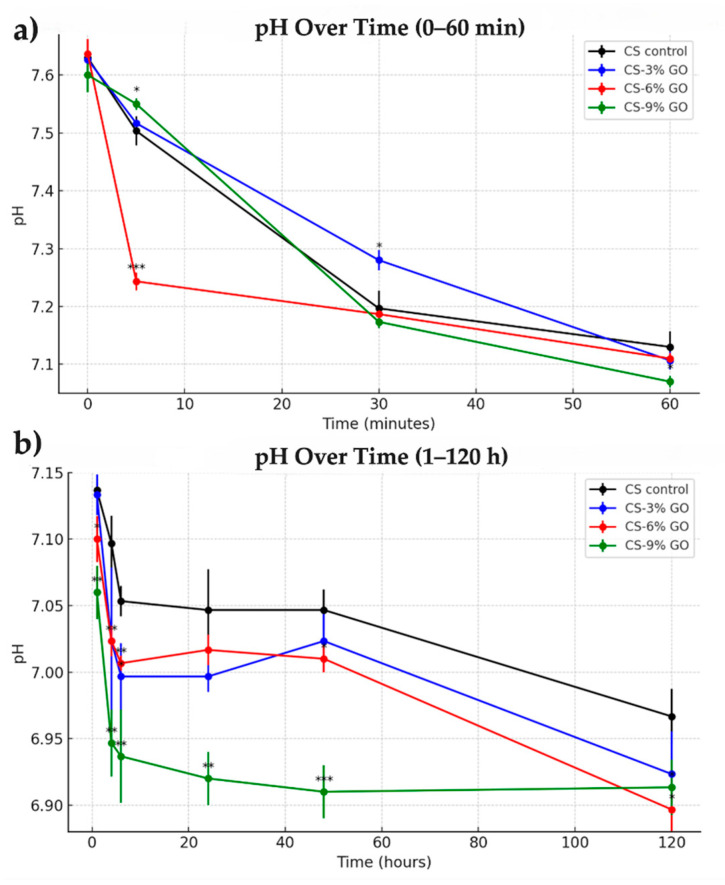
The pH behavior of CS-based films. (**a**) pH variation of CS and CS-GO scaffolds during the first 60 min of immersion in PBS; (**b**) pH variation of the same samples over extended immersion periods (1 to 120 h). Mean and standard deviation (SD) were calculated for each group at each time point. Statistical analysis was performed using one-way ANOVA, followed by Tukey’s post hoc test to compare groups. Statistical significance was denoted as follows: *p* < 0.05 (*); *p* < 0.01 (**); *p* < 0.001 (***). All experiments were performed in triplicate (*n* = 3).

All samples were introduced into a pure PBS solution with an initial pH of 7.62. As shown in Figure 8a, within the first 5 min, a rapid decline in pH was observed across all groups, most notably in the CS–6% GO scaffold, which dropped to 7.24. At 30 min, pH values converged, ranging from 7.19 (CS-6% GO) to 7.26 (CS-3% GO). By 60 min, pH had slightly decreased further for all groups, reaching 7.13 for CS control, 7.12 for CS-3% GO, 7.10 for CS-6% GO, and 7.06 for CS-9% GO. This trend suggested an initial equilibration phase, where GO incorporation, especially at higher concentrations, subtly lowered pH, likely due to the release of functional groups (e.g., –COOH) or ionic interactions within the scaffold matrix.

Over the long term (Figure 8b), all scaffolds exhibited a gradual decline in pH during the first 6 h, with the effect being more pronounced in those containing higher concentrations of GO, likely as a result of acidic functional groups released from the graphene oxide surface. From 6 to 48 h, the pH remained stable across all samples, indicating a period of equilibrium. After 48 h, a gradual decrease in pH was observed, with final values ranging between 6.89 (CS–6% GO) and 6.95 (CS control) at 120 h.

The stability of pH is crucial for neuroregeneration, as neural cells are sensitive to pH fluctuations [72]. The observed pH stabilization indicated that, after the initial interaction, these scaffolds might provide a favorable environment for neural cell growth and regeneration.

#### 3.7.2. Conductivity Variation in Solution

This experiment evaluated changes in ionic conductivity of CS and CS-GO scaffolds when immersed in PBS at neutral pH. The initial conductivity of the pure PBS solution was 15.97 mS/cm. The setup simulated the interaction of materials with a physiological solution, offering insights into their effects on the ionic environment for biomedical applications such as neuroregeneration.

**Figure 9:**
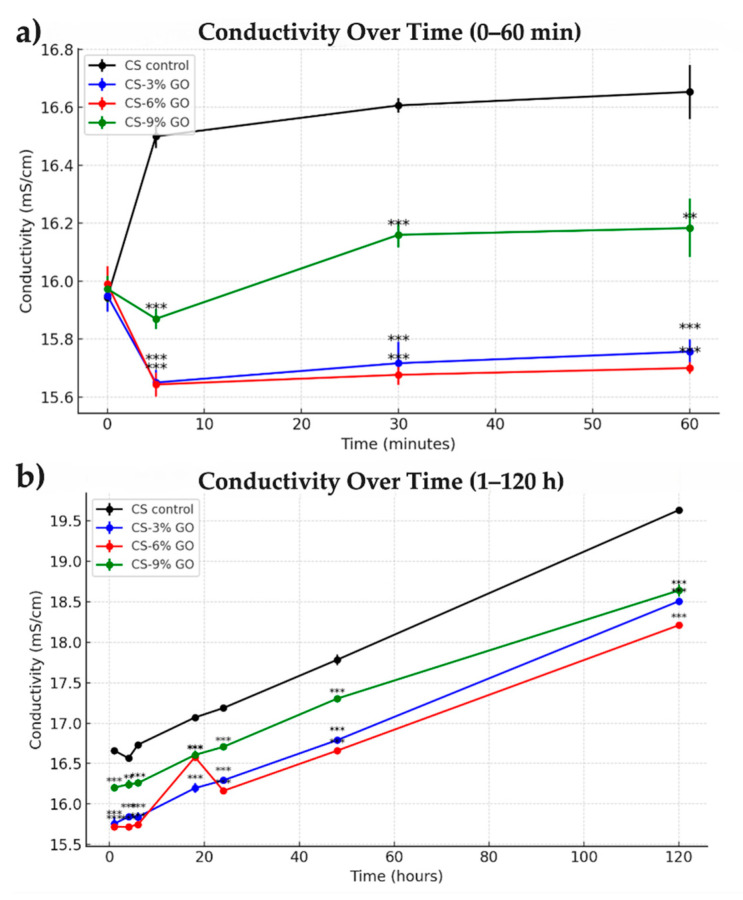
Ionic conductivity of CS and CS-GO scaffolds in PBS over short-term (**a**) and long-term (**b**) periods. Mean and standard deviation (SD) were calculated for each group at each time point. Statistical analysis was performed using one-way ANOVA, followed by Tukey’s post hoc test to compare groups. Statistical significance was denoted as follows: *p <* 0.01 (**); *p <* 0.001 (***). All experiments were performed in triplicate (*n* = 3).

As presented in Figure 9a, within the first 5 min, a sharp divergence in conductivity values was observed. The CS control scaffold demonstrated a marked increase to 16.47 mS/cm, while the GO-containing scaffolds showed a slight decrease, particularly CS–6% GO (15.59 mS/cm). This initial drop may have been attributed to the structural rearrangement or slight swelling delay caused by GO incorporation, which affected ion mobility. Over the next 30–60 min, a gradual increase in conductivity was observed across all samples. By 60 min, the conductivity values were as follows: CS control—16.66 mS/cm; CS-9% GO—16.20 mS/cm; CS-3% GO—15.73 mS/cm; CS-6% GO—15.71 mS/cm.

Extended conductivity measurements (Figure 9b) revealed progressive in ionic conductivity for all scaffolds over time, reflecting increased scaffold hydration and ion diffusion within the porous matrix. The CS control scaffold continued to exhibit superior conductivity throughout the observation period, increasing steadily from 16.66 mS/cm at 1 h to 19.64 mS/cm at 120 h. Among the GO-incorporated samples, CS-3% GO showed the most significant long-term increase, reaching 18.50 mS/cm, followed by CS-9% GO (18.58 mS/cm) and CS-6% GO (18.15 mS/cm).

In conclusion, both CS and GO-enhanced scaffolds interacted with the PBS solution, increasing conductivity over time. The lower conductivity in GO-enhanced scaffolds highlighted improved stability and controlled ion release, supporting their potential in neuroregeneration.

### 3.8. Electrochemical Conductivity Measurements of CS-GO

In the case of 3% and 6% CS-GO films, the relative conductivity values (dimensionless), calculated as the ratio of the considered conductivity over the lowest average conductivity value, increased marginally. This indicated a relative constancy of conductivity within the measurement errors for small concentration values of GO. This behavior was explained by the fact that, at low concentrations, small GO particles were completely surrounded by CS, having no physical contact points between them, thus limiting the formation of conductive pathways (Table 4).

**Figure 10:**
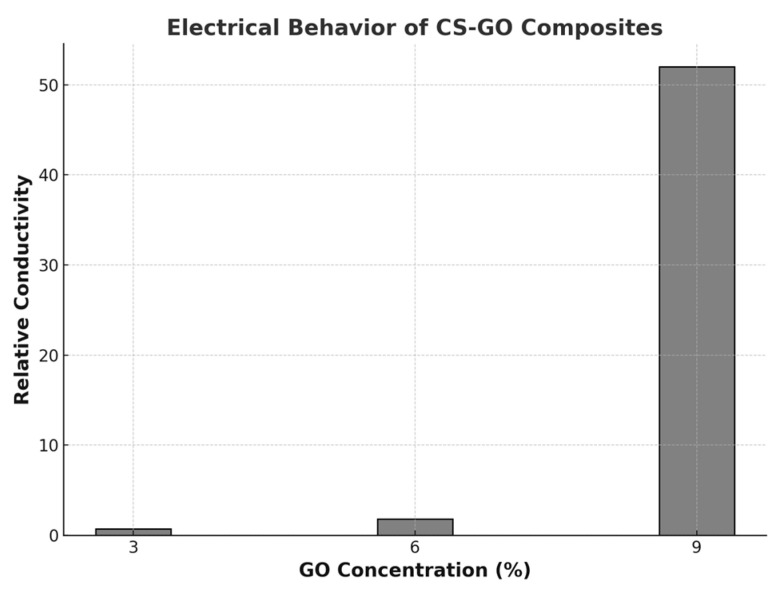
The relative conductivity values increased slightly for low GO concentrations (3% to 6%), while a sharp increase was observed above 6%. CS-GO scaffolds exhibit enhanced electrical conductivity with increasing GO concentration.

A critical concentration of GO was identified between 6% and 9%, where relative conductivity increased significantly, by approximately 52-fold compared to the minimum value, as shown in Figure 10. However, further studies are needed to optimize the concentration to ensure effective electrical stimulation and controlled release of biologically active agents under an applied electric field. Certainly, the conductivity of the native neural tissues can be considered golden standard, and according to the literature, values ranged from µS/cm to mS/cm [73].

### 3.9. SEM Analysis

**Figure 11:**
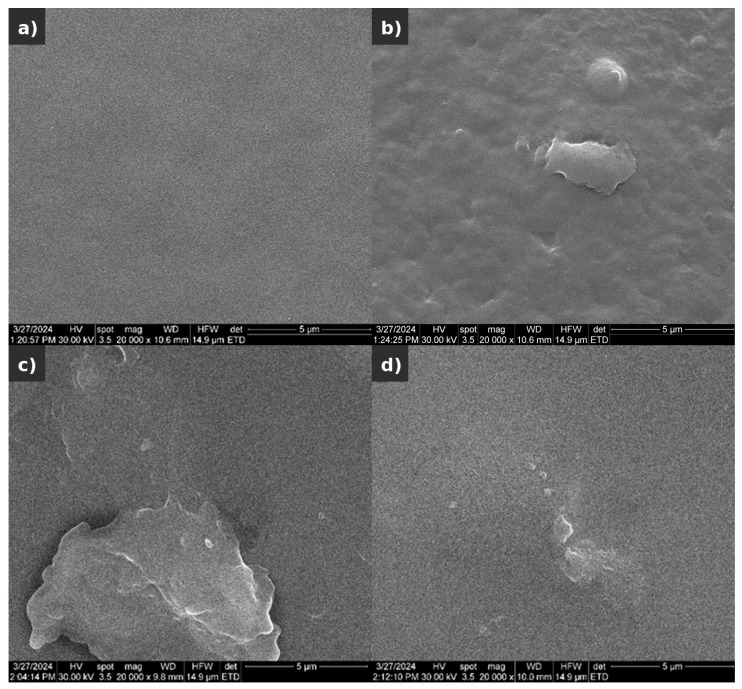
SEM analysis: (**a**) CS control, (**b**) CS-3% GO, (**c**) CS-6% GO, and (**d**) CS-9% GO.

In general, as observed in the visual examination, the surfaces of the samples were smooth with limited hill–valley structures (Figure 11). These distinctive features reflected a non-smooth morphology at the submicron level and were associated with the GO-derived heterogeneity at that scale [72]. These agglomerates varied in size and shape, reflecting the complex associations between the components, without being necessarily associated with large agglomerates of GO [74]. The pure CS scaffold (Figure 11a) displayed a smooth and uniform surface with minimal irregularities or protrusions, showing only a minor hill–valley aspect as observed from the variation in the color (black intensity). This smoother surface morphology of pure CS might not have been optimal for cell adhesion and proliferation compared to more textured surfaces. However, CS’s natural biocompatibility still supported cellular attachment [75]. Modifications or the incorporation of additional materials, such as GO, was considered necessary to enhance cell adhesion and interaction for better neuroregenerative outcomes.

Most probably, the jagged edges of GO sheets and agglomerates formed during oxidation process had a strong influence on the surface defects (Figure 11b–d). Agglomerates and clusters of GO stacked on top of each other, especially at higher concentrations. The CS-3% GO (Figure 11b) scaffold showed a more textured surface with visible inclusions of GO compared to the pure CS scaffold. While the overall distribution of GO remained generally uniform, localized agglomerates were observed, indicating areas of higher concentration. The CS-6% GO scaffold displayed an even more pronounced textured surface. The increased presence of GO led to a noticeable increase in surface roughness compared to the pure CS scaffold. The scaffold maintained its structural integrity and exhibited enhanced mechanical properties. The CS-9% GO scaffold exhibited a smoother surface with less pronounced texturing than the lower GO concentration samples. Although some GO agglomerates remained visible, the overall surface appeared more compact and homogeneous. The scaffold’s porosity decreased with higher GO content, potentially affecting its overall performance in tissue engineering applications.

### 3.10. Biocompatibility Assessment of SH-SY5Y Cell Culture in Contact with CS-GO Scaffolds

**Figure 12:**
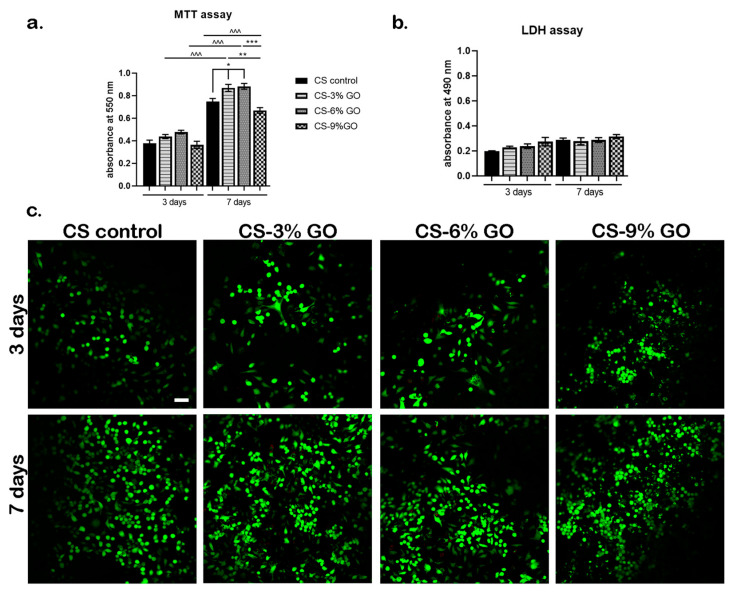
In vitro biocompatibility investigations of CS-GO scaffolds in contact with SH-SY5Y cells during one week of culture. (**a**) Cell viability and proliferation profiles as revealed by the MTT assay. Statistical significance: * *p* < 0.05; ** *p* < 0.01; ^^^ and *** *p* < 0.001; (*) was used to compare different composites during the same time point of testing and (^) was used to compare the same material at different time points during the experiment. (**b**) CS-GO-enriched scaffold toxicity evaluated by the LDH assay. (**c**) Fluorescence microscopy assessment of live (green-labeled) and dead (red-labeled) SH-SY5Y cells in contact with CS-GO-enriched materials. Scale bar: 50 µm.

The biological analyses of CS-GO scaffolds are presented in Figure 12.

The cell viability and proliferation profile, as it resulted after performing the MTT assay (Figure 12a), revealed an overall good interaction between the SH-SY5Y cell culture and the tested CS-GO composites. After 3 days of culture under standard culture conditions, cell viability was slightly different between the materials, but with no significant difference. After 7 days of in vitro cell culture, a significantly higher (*p* < 0.05) viability rate was registered on CS-GO 3% and 6% compared to plain CS control. Furthermore, viability on CS-GO 9% scaffold cells was significantly lower compared to CS-GO 3% and CS-GO 6% (*p* < 0.01 and *p* < 0.001, respectively). From 3 to 7 days of culture, SH-SY5Y proliferated significantly (*p* < 0.001) on all tested materials, indicating that the scaffolds provided an optimal environment for proper cell growth.

The LDH assay assessed the cytotoxic potential of CS-GO enriched materials in contact with SH-SY5Y cells (Figure 12b). The results revealed relative low levels of LDH present in the culture media after 3 days, with no significant differences between the composites. The levels remained constant during one week of in vitro cell culture, without causing significant toxicity upon SH-SY5Y cells. A slightly higher level of LDH was obtained in the presence of the CS-GO 9% scaffold, but not at a statistically significant level.

Live/Dead fluorescence microscopy (Figure 12c) corroborated the MTT and LDH assays results. At 3 days post-seeding, cells were observed to adhere on the surface of the materials. Moreover, on CS-GO 3% and CS-GO 6%, cell shape was elongated, whereas on CS control, there was little-to-no cell elongation on the surface. On the CS-CO 9% composite, cells were labeled in green, but at the same time, they did not present a characteristic shape, revealing that the culture is under stress. After 7 days of in vitro cell culture, images revealed a significant increase in cell density, confirming that cells proliferated in the presence of CS-GO materials. Cells formed clusters, indicating positive cellular interaction with the surface of the materials, which in turn supported viability and growth. On CS control, SH-SY5Y cells presented an elongated phenotype, a behavior also noticed in the presence of CS-GO 3% and CS-GO 6%. In contrast, on CS-CO 9%, cells were not encouraged to elongate and displayed a rounded shape.

GO is a widely studied carbon-based nanomaterial for various applications in tissue regeneration. Due to its tunability and electro-conductivity properties, it is considered as a major candidate for nerve regeneration [76,77,78]. Our results highlight a good biological interaction between SHSY-5Y cell cultures and CS-GO enriched scaffolds, with an emphasis on CS-GO 3% and CS-GO 6%, which demonstrated the best outcomes. The presence of more GO (9%) did not necessarily increase the biological response in this experimental setup. Therefore, for SH-SY5Y cells, the optimal concentration of GO is up to 6%. Similar results were obtained in another study [79], where the same cells were tested for their biocompatibility and differentiation potential in the presence of a GO and silver nanoparticle composite. Thus, the newly developed CS-GO scaffolds are biocompatible with SH-SY5Y cells and can be validated as candidates for nerve regeneration purposes. At 3 and 6% GO, the biocompatibility significantly increased compared to pure CS, but at 9% GO, the viability slightly decreased (but not statistically significantly, as seen in Figure 12a).

## 4. Conclusions

This study reported the development of CS-GO composite films with enhanced physicochemical and biological performance. FTIR analysis confirmed strong chemical interactions between CS and GO, supporting successful integration into the polymer matrix. Electrical conductivity remained stable up to 6% GO, after which a sharp increase was observed, indicating a critical concentration for tuning electrical properties. Morphological characterization showed well-distributed, textured surfaces at 6% GO, favorable for cell attachment. Biological assays demonstrated that CS-GO films with 6% GO supported optimal cell viability and proliferation without inducing cytotoxic effects. The high loading of GO and the high viability can be explained considering the strong interaction between the phases and the fact that no free GO particles are released and induce toxicity. This composition emerged as the most effective in balancing structural, electrical, and biological properties. Thermal analysis revealed improved stability, with higher degradation temperatures in GO-containing films, while swelling tests showed increased fluid uptake with GO concentration—an essential feature for tissue engineering.

Overall, the incorporation of GO significantly improved CS film performance, with 6% GO identified as the optimal formulation for neuroregenerative applications. Further studies should explore the mechanical properties, electric stimulation capability, loading and release of neuroregenerative agents, and in vivo behavior on small animals, following the imposed ethical procedures.

## Figures and Tables

**Table 1:** Sample codes, descriptions, and the mass of GO added for each sample. In all cases, a 30 mg/mL CS solution was used.

Sample Code	Description	GO (mg/mL)
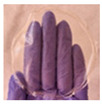 CS control	The first image (a) shows a plain CS scaffold without any enhancements. It appears translucent and flexible, exhibiting the typical characteristics of a pure CS-based material.	0.0
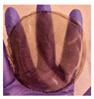 CS + 3%GO	The second image (b) displays a CS scaffold enhanced with 3% GO. The scaffold appears slightly darker and less translucent than the simple CS scaffold, indicating the incorporation of the enhancing agent.	0.92
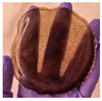 CS + 6%GO	The third image (c) features a CS scaffold enhanced with 6% GO. This scaffold is noticeably darker and more opaque, suggesting a higher additive concentration.	1.91
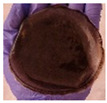 CS + 9%GO	The last image (d) presents a CS scaffold enhanced with 9% GO. The scaffold is the darkest and opaquest, reflecting the highest enhancement level.	2.96

**Table 2:** Measured thickness and calculated film opacity.

Sample Code	CS	CS_GO3%	CS_GO6%	CS_GO9%
Thickness (mm)	0.42 ± 0.02	0.45 ± 0.01	0.44 ± 0.02	0.49 ± 0.03
Opacity	0.265 ± 0.012 ^a^	1.812 ± 0.040 ^b^	2.563 ± 0.111 ^c^	2.799 ± 0.172 ^c^

Different superscript letters indicate statistically significant differences between films (*p* < 0.05). All experiments were performed in triplicate (*n* = 3).

**Table 3:** Principal data from thermal analysis.

Sample	Mass Loss (%) RT-200 °C	Endothermic Effect (^o^C)	Mass Loss (%) 200–400 °C	Residual Mass (%)	T_5%_	T_10%_	T_15%_
CS	9.41%	125.0 °C	40.75%	22.57%	138 °C	207 °C	236 °C
CS_GO3%	11.73%	112.1 °C	38.10%	7.33%	125 °C	181 °C	218.5 °C
CS_GO6%	10.10%	116.0 °C	39.44%	8.16%	133 °C	199 °C	228 °C
CS_GO9%	10.70%	117.0 °C	38.14%	6.48%	139 °C	193 °C	227.5 °C

**Table 4:** Calculation of the relative conductivity for the three concentrations (3%, 6%, and 9%) of GO. At all concentrations, the relative conductivity values increased marginally. All experiments were performed in triplicate (*n* = 3).

GO Concentration (%)	Average Conductivity Ω^−1.^cm^−1^	Relative Standard Deviation	Relative Conductivity *
3	5.07 × 10^−11^	3.12 × 10^0^	1.00 × 10^0^
6	1.03 × 10^−10^	6.80 × 10^0^	2.03 × 10^0^
9	2.64 × 10^−9^	5.39 × 10^0^	5.20 × 10^1^

## Data Availability

The original contributions presented in this study are included in the article/Supplementary Materials. Further inquiries can be directed to the corresponding author.

## Supplementary Materials

The following supporting information can be downloaded at: https://www.mdpi.com/article/10.3390/polym17172398/s1, Detailed data on the DSC curve in Figure 6 are provided in Supplementary Materials.

## Abbreviations

The following abbreviations are used in this manuscript:

ATR	Attenuated Total Reflectance
CO_2_	Carbon Dioxide
CS	Chitosan
d	Thickness of the Deposited Layer
DSC	Differential Scanning Calorimetry
EDS	Energy Dispersive X-ray Spectroscopy
FBS	Fetal Bovine Serum
FEG	Field Emission Gun
FTIR	Fourier-Transform Infrared Spectroscopy
GO	Graphene Oxide
λ	Electrical Conductivity
LDH	Lactate Dehydrogenase
LIVE/DEAD	Live/Dead Cell Viability Assay
MEM	Minimum Essential Medium (Eagle)
MTT	3-(4,5-dimethylthiazol-2-yl)-2,5-diphenyltetrazolium bromide
NGCs	Nerve Guidance Conduits
PBS	Phosphate-Buffered Saline
PL	Photoluminescence
R	Electrical Resistance (Ohm)
SEM	Scanning Electron Microscopy
SH-SY5Y	Human Neuroblastoma Cell Line
S	Cross-sectional Area
TG	Thermogravimetry
TG-DSC	Thermogravimetric Analysis–Differential Scanning Calorimetry
UV-Vis	Ultraviolet–Visible Spectroscopy
