# Supplementary figures and images for: Conductive Chitosan–Graphene Oxide Scaffold with Applications in Peripheral Nerve Tissue Engineering
Source: Polymers (Basel). 2025 Sep 2;17(17):2398. doi: 10.3390/polym17172398 (PMC12431565; doi:10.3390/polym17172398)

# Supplementary Materials

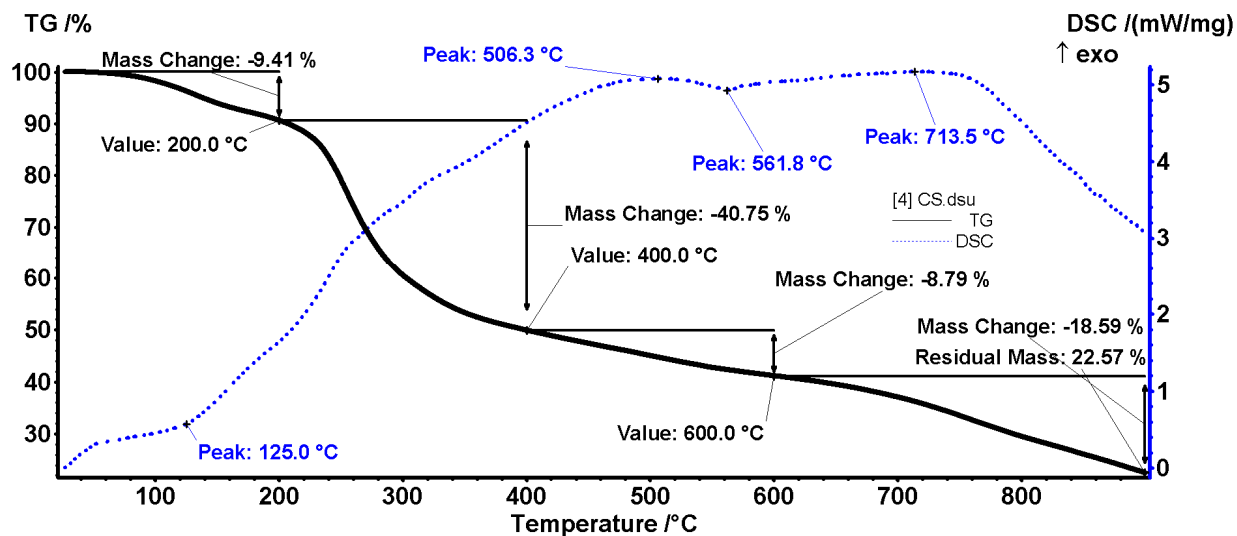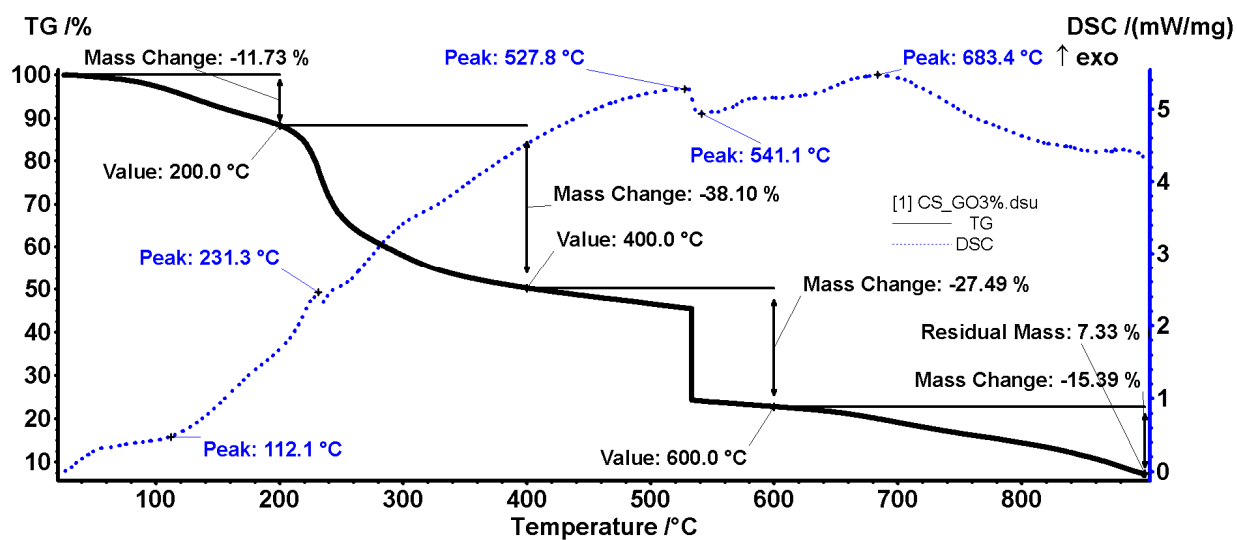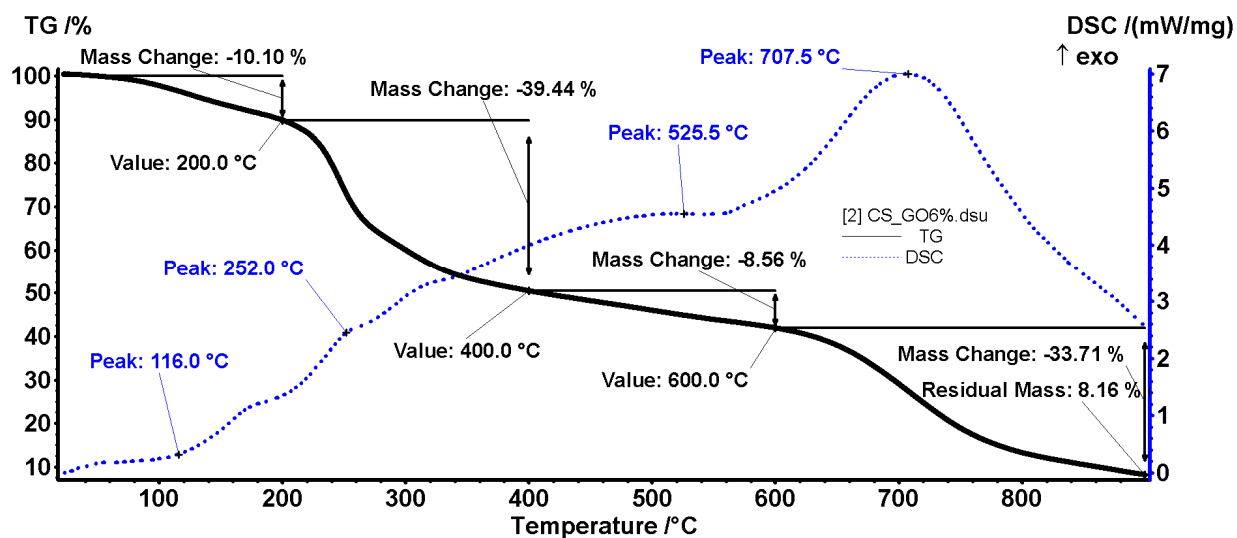

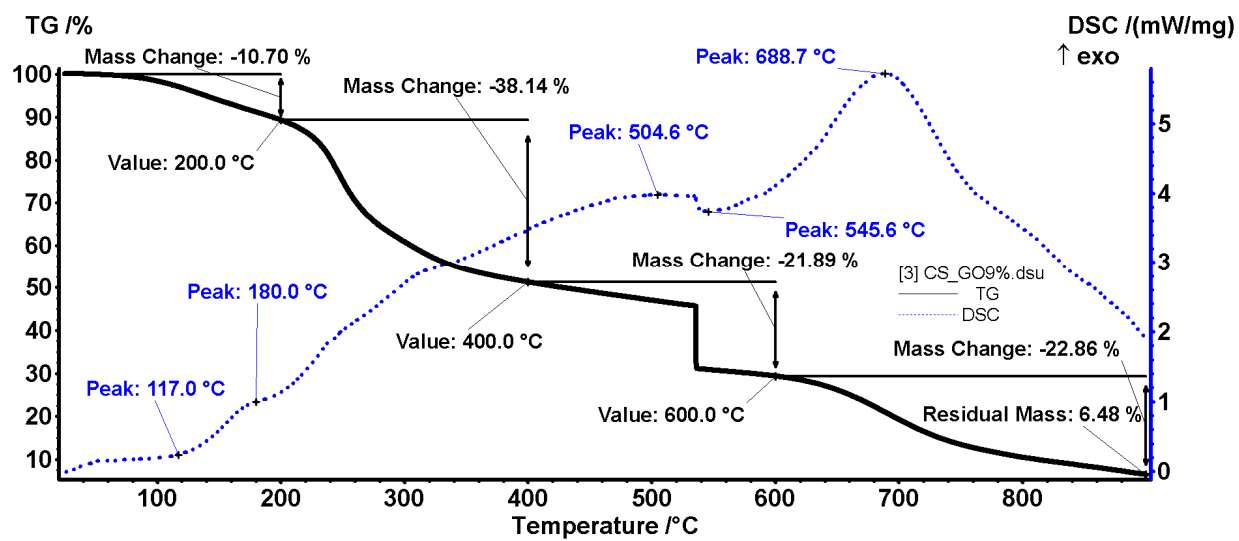

Supplement: Supplementary file 1 [file polymers-17-02398-s001.zip › polymers-3784622-supplementary.pdf]
